# Winged Scapula: A Comprehensive Review of Surgical Treatment

**DOI:** 10.7759/cureus.1923

**Published:** 2017-12-07

**Authors:** Marc Vetter, Ordessia Charran, Emre Yilmaz, Bryan Edwards, Mitchel A Muhleman, Rod J Oskouian, R. Shane Tubbs, Marios Loukas

**Affiliations:** 1 Seattle Science Foundation; 2 Department of Anatomical Sciences, St. George's University School of Medicine, Grenada, West Indies; 3 Swedish Medical Center, Swedish Neuroscience Institute; 4 Neurosurgery, Swedish Neuroscience Institute; 5 Neurosurgery, Seattle Science Foundation

**Keywords:** winged scapula, winging of scapula, accessory nerve, long thoracic nerve, trapezius, serratus anterior, scapulopexy, eden-lange method

## Abstract

Winged scapula is caused by paralysis of the serratus anterior or trapezius muscles due to damage to the long thoracic or accessory nerves, resulting in loss of strength and range of motion of the shoulder. Because this nerve damage can happen in a variety of ways, initial diagnosis may be overlooked. This paper discusses the anatomical structures involved in several variations of winged scapula, the pathogenesis of winged scapula, and several historical and contemporary surgical procedures used to treat this condition. Additionally, this review builds upon the conclusions of several studies in order to suggest areas for continued research regarding the treatment of winged scapula.

## Introduction and background

In 1723, Winslow published the first description of scapular winging [1-2]. The most common etiology of winged scapula is serratus anterior palsy caused from traumatic or iatrogenic damage to the long thoracic nerve [3-5]. Less frequently observed is a winged scapula resulting from trapezius palsy. This type of palsy is caused by traumatic or iatrogenic damage to the accessory nerve [4, 6]. For surgeons, awareness of potential variations of the long thoracic nerve and the accessory nerve can decrease the incidence of winged scapula with an iatrogenic cause [7-10].

Several surgical procedures have been used in the past to treat winged scapula. In 1904, Alfred Tubby was the first to describe the surgical procedure known as a pectoralis major transfer. The pectoralis major transfer and split pectoralis major transfer procedures involve transposition of the pectoralis major muscle from the bicipital groove of the humerus and the medial portion of the clavicle to the scapula, thus reestablishing the shoulder’s range of motion [11]. The Eden-Lange procedure, described in 1924, involves the transfer of the tendon of the levator scapulae muscle to the acromion and the attachment of the rhomboid muscles to the central part of the scapula. This technique aims to reproduce the functionality lost by trapezius muscle paralysis [12]. The modified Eden-Lange procedure is similarly performed to correct winged scapula due to trapezius muscle palsy. It involves transferring the rhomboid minor to the supraspinatus fossa while displacing the rhomboid major to the infraspinatus fossa. In addition, the levator scapulae is attached to the spine of the scapula [4].

More recent surgical procedures used to treat winged scapula include scapulothoracic arthrodesis, in which the scapula is fixed to the third through sixth ribs using a semitubular fragment plate [13]. This procedure is less commonly utilized compared to a scapulopexy without arthrodesis, which is essentially the same procedure but without the use of the fragment plate, thus directly fusing the scapula and ribs [14]. Additional procedures can include microneurolysis of the long thoracic nerve, nerve transfer, and nerve grafting [5, 15-16].

## Review

Anatomy

The scapula, a flat, triangular bone that contributes to the posterior aspect of the shoulder girdle, is found bilaterally on the dorsal surface of the body and is stabilized through the actions of both the serratus anterior and the trapezius muscles [17].

The serratus anterior muscle originates along the anterolateral region of the first eight ribs, winds around the chest wall, and inserts along the deep side of the ventral border of the scapula [3]. This muscle produces an upward rotation and protraction of the scapula during motion and also functions as an accessory muscle during respiration [18]. The long thoracic nerve innervates the serratus anterior muscle [9].

The long thoracic nerve originates from the upper part of the superior trunk of the brachial plexus, although variations have been reported [7-8]. This nerve is generally composed of cervical nerves C5, C6, and C7 but compositional variations can exist such that C5 or C7 may not contribute or C8 may join the other three nerve roots to collectively become the long thoracic nerve [7-8]. In most circumstances, the ventral rami of C5 and C6 traverse the middle scalene muscle where they merge before joining the C7 root. The anterior branches of these nerve roots fuse before passing posterior to the brachial plexus to give rise to the long thoracic nerve [19]. The long thoracic nerve then runs posterior to the clavicle and anterior to the first rib before continuing distally as far as the eighth or ninth rib to innervate the serratus anterior muscle [3, 9]. Alternatively, although far less common, the fusion of the cervical nerves can occur over the first rib, without passing through the scalenus medius [7].

The upper digitations of the serratus anterior muscle may be innervated directly by C4 or C5 independently, without these nerves merging to form the main trunk of the long thoracic nerve [7-8]. There are also variations in the relation of the long thoracic nerve to the middle scalene muscle, as the different roots of this nerve may lie posterior, anterior, or even pass through the muscle [8]. Thoracic surgeons should note these variations in the origin and course of the long thoracic nerve since iatrogenic injury of this nerve is a common cause of scapular winging [9-10, 4].

The long thoracic nerve has a relatively smaller diameter than the other nerves of the brachial plexus. Additionally, the long thoracic nerve is surrounded by less connective tissue and travels over the superficial surface of the serratus anterior muscle, thus it is more exposed along its course [5]. These factors, coupled with the winding course of the long thoracic nerve, may account for the nerve’s susceptibility to injury [3].

The trapezius acts on the scapula to aid elevation, retraction, and rotation. It inserts on the acromion, spine of the scapula, and lateral third of the clavicle from its origin at the posterior midline of the thorax along the cervical and thoracic spine. The accessory nerve innervates the trapezius muscle by coursing just below the superficial fascia as it passes along the floor of the posterior cervical triangle on its way to insert in the deep medial half of the muscle. Like the long thoracic nerve, the accessory nerve is very superficial in the neck and is therefore more susceptible to injury [3].

Pathogenesis

A winged scapula is evident when the vertebral border or inferior angle of the scapula displays unusual prominence [20, 3]. This condition results in limited range of motion and decreased shoulder strength. Furthermore, patients experience discomfort and cosmetic deformity [4]. Primary winged scapula is caused by neurologic injury, detrimental changes in the bone, or anomalies of periscapular soft-tissue [21]. Secondary winged scapula manifests due to glenohumeral and subacromial conditions in which correction of these conditions leads to winged scapula [21].

Nerve damage can result from compressive injury due to anesthesia or a condition known as Saturday night palsy, which can damage the long thoracic or accessory nerves. Both types of damage may lead to scapular winging [3]. Also, heavy load bearing can cause nerve entrapment with resultant atrophy of the trapezius and serratus anterior and thus winging of the scapula [6]. Surgical procedures in the thoracic region such as radical mastectomy, resection of the first rib, and transthoracic sympathectomy [22, 4] can expose the long thoracic nerve and make it susceptible to damage. It was reported that the long thoracic nerve could be damaged in 30% of infraclavicular procedures involving surgical dissection in the axilla [5].

Other procedures such as a posterolateral thoracotomy incision for closed heart procedures in infants and children place the long thoracic nerve at risk due to the division of the latissimus dorsi and serratus anterior muscles. After this procedure, winged scapula was reported in 77% of cases while complications such as thoracic cage deformity and elevation of the shoulder were only reported in 18% and 61% of cases, respectively [23]. Athletic activity and overuse can cause nerve traction and give rise to winged scapula [3]. Additional causes of nerve injury leading to winged scapula can include electric shock, cold exposure or blunt injury, viral infection, immunization, and brachial neuritis [3]. Subscapular osteochondroma [21, 24] and instability of the scapulothoracic joint [14] can also lead to the development of winged scapula [21, 24].

Prognosis

It is possible for some patients suffering from scapular winging to recover most or all functionality of the shoulder joint [3]. However, if left untreated, several consequences can ensue including adhesive capsulitis, subacromial impingement, and brachial plexus radiculitis [5-6].

The recommended treatment for winged scapula is initially non-surgical, involving conservative measures such as physical therapy. Varied results have been achieved using braces, standard arm slings and other orthotics [3, 25]. No treatment method has been experimentally confirmed to be favored. However, some propose that non-operative treatment may be the best course of action for patients with minimal symptoms as well as older and more sedentary patients [4].

Examples of two unique clinical cases

A case of winged scapula was reported to have occurred upon rupture of the rhomboidius major muscle and was made worse by the thinning of the lower thoracic bundle of the trapezius [21]. The muscles were repaired, and reefing was performed via an incision made parallel to the vertebral border of the scapula. Upon muscle fixation, the scapula was stabilized to the chest wall. The patient recovery was supplemented with physical therapy and after two months, no obvious weakness, deformity, or pain was noted and limited range of motion was intact.

Another unique case was described that featured a 17-year-old male with bilateral winged scapula - the result of peripheral nerve palsy caused by Lyme disease contracted six years prior to presentation [26]. Electrophysiology studies indicated irreversible loss of both long thoracic nerves [26]. After repeated attempts at physiotherapy to no avail, the patient underwent operative stabilization of the scapula. This procedure involved preparation of a strip of fascia lata which was folded twice lengthwise and secured. Surgeons used a semicircular approach underneath the axilla from the coracoid to the tip of the scapula. The sternocostal segment of the pectoralis muscle was detached with a periosteal sleeve from its insertion on the humerus. The graft of fascia was then attached to the end of the pectoralis tendon, and then passed under the latissimus dorsi, directly over the paralyzed serratus anterior muscle to the lower pole of the scapula. An 8 mm hole was drilled at the tip of the scapula through which the graft traversed and looped around the axillary border. The folded section of the fascia lata graft was connected at the junction of the fascia to the serratus anterior. Full restoration of shoulder function was achieved three months postoperatively [27].

The evolution of surgical treatment for winged scapula

One of the contemporary procedures used to treat winged scapula secondary to trapezius palsy is scapulothoracic arthrodesis [4]. This surgical procedure evolved from another standard technique known as the Eden-Lange procedure, which involves the transfer of the tendon of the levator scapulae muscle to the acromion and the attachment of the rhomboids to the central part of the scapula [12]. In 2008, the Eden-Lange procedure was modified to instead transfer the rhomboid minor to the supraspinatus fossa and the rhomboid major to the infraspinatus fossa. In addition, the levator scapulae was attached to the spine of the scapula [4].

In situations of serratus anterior palsy, the split pectoralis major transfer without fascial graft is commonly utilized. This technique involves the transposition of the sternal head of the pectoralis major from its humeral insertion to the inferomedial portion of the scapula [4]. New techniques involving nerve transfer and nerve grafting are becoming more popular today. Several feasibility studies providing crucial postoperative details such as success and complication rates for these procedures are described below.

Pectoralis Major Transfer

This procedure involves transferring the insertion of the sternal part of the pectoralis major to the lower pole of the scapula. This muscle transfer technique allows for dynamic control and potentially normal scapulothoracic movement. Furthermore, this muscle placement most closely mimics the natural function of the serratus anterior, making this technique a reasonable candidate for surgical correction of winged scapula [11].

The pectoralis major transfer technique requires the patient to be supine with the scapula displaced laterally and the arm abducted. The incision is made lateral to the insertion of the pectoralis major muscle and it continues posteriorly across the axillary floor toward the lower pole of the scapula. The V-shaped fold of the tendon of the sternal portion of the pectoralis major muscle is detached near the humerus so that sufficient tendon is obtained for attachment to the tube of fascia lata.

The now-free end of the pectoralis major tendon is inserted into the fascial tube and anchored. After the supraspinatus and infraspinatus muscles are retracted, a 6 mm hole is drilled through the scapula bone, around 2.5 cm above the lower pole and 1.3 cm from the axillary border. The fascial tube is passed through this aperture, tightened, and secured. The lower branch of the subscapular nerve is identified and protected during this procedure. After the surgery, the shoulder is immobilized for three weeks in a 15° abduction pillow. Following this period, passive range of motion exercises, limited to 30° of flexion and 80° of internal rotation, are undertaken for six weeks [28-29].

Although nine out of 15 patients who underwent the pectoralis major transfer had successful results, they still experienced minor weakness in shoulder abduction [11]. Two patients reported fair results, as they had variable and incomplete control of winging during normal active movement. Four patients required a second operation to rectify the failure of the first procedure. In one instance, the two tails of the fascia lata extension became frayed at the insertions. In other cases, the fascia lata extension had been replaced by scar tissue that prevented contraction of the transferred muscle. This technique proved to have mixed results and it is difficult to predict the potential sequelae in different cases [11].

Split Pectoralis Major Transfer

The split pectoralis major transfer technique involves the transposition of the sternal head of the pectoralis major from its humeral insertion to the inferomedial region of the scapula and is used for the treatment of winged scapula secondary to serratus anterior palsy [4]. The patient maintains a lateral decubitus position with the arm abducted to emphasize the hiatus between the sternal and clavicular heads of the pectoralis major. The first incision is made along the axillary skin crease for 5 cm and the second incision made over the inferior angle of the scapula for 6 cm. Sharp dissection of the sternal head away from its insertion on the humerus to the axilla is carried out medially against the chest wall to avoid lateral neurovascular structures. The cut end of the tendon of the pectoralis major is then shuttled through the second incision. Once the scapula is orientated with the tendon in position, sutures are passed through the drill holes in the scapula. Upon routine closure, patients find that the resultant scar is not cosmetically deforming. Most patients regained full range of motion upon recovery from the procedure [4].

Eden-Lange Procedure

The Eden-Lange procedure involves the transfer of the tendon of the levator scapulae muscle to the acromion and the attachment of the rhomboids to the central part of the scapula within the infraspinatus fossa [12]. Eden (1924) described this technique for treatment of iatrogenic trapezius palsy [30]. Two patients with paralysis of the trapezius muscle reported pain relief and restoration of shoulder function after this procedure [12]. Other studies also report favorable results using this procedure [31-32, 12].

Modified Eden-Lange Procedure

The modified Eden-Lange procedure is also used to correct winged scapula caused by trapezius palsy [4]. In this case, trauma to a branch of the accessory nerve innervating the trapezius precludes stabilization of the scapula. This surgery is performed with the patient in the full lateral decubitus position with a 15° elevation of the head of the bed. A 10 cm incision from the superior edge to the inferior angle of the scapula is created between the spinous processes and the medial border of the scapula. The arm is manipulated to expose the levator scapulae and the major and minor rhomboids. A small piece of osteotomized bone with its tendinous insertions is detached. Alternatively, the tendons alone can be detached. The rhomboid minor is transferred into the supraspinatus fossa upon elevation of the supraspinatus muscle. Similarly, the rhomboid major is displaced into the infraspinatus fossa once the infraspinatus is elevated. Drill holes in the respective fossae are used to suture the muscles into place. The second incision is opened over the spine of the scapula to facilitate transfer of the levator scapulae. The levator scapulae is passed through a tunnel made through the trapezius and sutured to the spine of the scapula via three drill holes [4]. In one particular study, this technique was performed on six patients with patients reporting postoperative satisfaction and no subsequent winging of the scapula [4].

Scapulothoracic Arthrodesis

This is a less common approach to treatment of winged scapula [5]. In a 2005 study, Krishnan, et al. analyze the outcome of scapulothoracic arthrodesis performed on 22 patients (24 shoulders) to rectify scapular winging. A plate and wire fixation approach was used in the fusion of the medial border of the scapula with an autologous bone graft. An incision was made along the medial border of the scapula from the scapular spine to the inferior angle. The trapezius was retracted medially and the rhomboid muscles were elevated. Next, the medial scapular border was retracted from the rib cage. Almost 33% of the serratus anterior and subscapularis muscles were resected off the anterior surface of the scapula to allow for a wide fusion surface. The subscapularis was not retracted past the midline of the scapula in order to prevent its denervation. The anterior surface of the scapula was roughened with a burr. However, the medial border was not thinned excessively to prevent fracture during hardware fixation [13].

The periosteum was stripped off each identified rib (third to sixth) adjacent to the decorticated anterior surface of the scapula. The ribs were roughened with the burr to remove all areas of soft tissue and thus maximize bony contact between the scapula and ribs. The lung in proximity to the operating field was deflated, and then cerclage wire was wound around each of the exposed ribs at the level where the scapula was to rest. Next, a semitubular large fragment plate with five or six holes was aligned on the posterior aspect of the medial border of the scapula and a 3 mm burr was used to create holes corresponding to those of the plate. The wires were threaded through the scapula and semitubular plate with the bone graft from the posterior iliac crest between the scapula and ribs. Tightening of the wires occurred with the scapula externally deviated 20°-25° from the midline. The semitubular plate facilitated uniform stress distribution along the bone [13].

Once this was complete, the lung was re-inflated and assessed for pneumothorax. The rhomboid muscles were fixed to their point of attachment and the incision closed. A chest tube was placed to treat pneumothorax and to allow drainage of reactive pleural effusion. The arm was maintained in neutral rotation and immobilized for 12 weeks [13].

In this method 91% of subjects experienced less shoulder pain and greater range of motion after the surgery. Some complications of this procedure included hardware failure, pulmonary sequelae, pseudarthrosis, and persistent pain. More than half of the patients in the study experienced one or more of these sequelae. This significantly high complication rate makes this procedure less favorable. It should be performed only when patients have had considerable preoperative counseling and are certain that they can no longer live with this condition [13].

Scapulopexy without Arthrodesis

A retrospective study of 13 patients with bilateral winged scapula secondary to facioscapulohumeral muscular dystrophy was analyzed to determine the effectiveness of scapulopexy. This technique involved repositioning and fixation of the scapula to four ribs using metal wires without performing arthrodesis. Patients were placed in a prone position and both shoulders were repaired [14].

Giannini, et al. (2007) began this procedure with a linear oblique incision on the medial border of the scapula through which the trapezius muscle was sectioned. The atrophic and fibrotic rhomboid major and minor and levator scapulae were detached from the vertebral border of the scapula. Other muscles such as the supraspinatus, infraspinatus, subscapularis, and serratus anterior were liberated from the vertebral border of the scapula.

A segment of the medial border of the scapula was exposed and the bone was displaced medially and caudally to expose the underlying ribs. Four ribs starting from the fourth or fifth were subperiosteally exposed and double metal wire was threaded under each rib. When the scapula was superimposed on the rib cage in its definitive position, four holes were drilled 1.5 cm from the medial border of the scapula opposite the prepared ribs. The metal wires were passed through these holes, then twisted and tightened until there was contact between the scapula and ribs. Once the wires were cut, the muscles were replaced over the posterior surface of the scapula before the wound was closed [14].

The limb was immobilized for six weeks. Once tolerated, isometric deltoid exercises and active exercises were performed with the goal of strengthening the glenohumeral joint muscles and reestablishing range of motion. It was reported that all patients were satisfied with the results of the procedure especially since there was improvement in the active range of motion of the shoulder. There was complete resolution of winged scapula and a favorable cosmetic outcome using this technique. In all 26 instances of scapulopexy, there was stable and enduring fixation of the scapula to the rib cage [14].

Microneurolysis of the Long Thoracic Nerve

Microneurolysis and decompression of the long thoracic nerve was used by Nath, et al. [5] to treat winged scapula in a study of 47 patients. Many of the subjects used in this study may have compressed the nerve in question due to contractions of the middle scalene muscle secondary to exercise. All patients participated in regular physical therapy prior to surgery.

In order to present the appropriate area for surgery, patients assumed a lawn-chair position using a shoulder roll, with the head and neck abducted away from the surgical field. An incision was made two finger-widths posterior and parallel to the clavicle. It extended 6-8 cm, flanking the lateral clavicular border of the sternocleidomastoid muscle. Although the platysma muscle was dissected through, the supraclavicular nerves beneath were avoided. The omohyoid muscle was retracted to reveal the scalene fat pad, which was also elevated to visualize the brachial plexus. In order to avoid iatrogenic injury, it is crucial at this point to identify the suprascapular branch of the upper trunk of the brachial plexus, which usually traverses the middle layer of the scalene fat pad [5, 33].

It is common to encounter epineural scarring at the trifurcation of the upper trunk. External neurolysis was accomplished using microsurgical instruments and techniques. The most superficial fibers of the anterior scalene muscle were resected so that only about 20% of the thickness of this muscle was diminished overall [5, 33].

The isolated long thoracic nerve was the subject of internal and external neurolysis. Internal neurolysis was executed as a rule in all cases. Resection of the middle scalene muscle allowed for decompression of the long thoracic nerve, especially toward the point of exit from the muscle. The epineurium of the nerve bore signs of surface neovascularization and narrowing at the site of nerve compression. Any fascial bands that were perceived to potentially compress the upper trunk and long thoracic nerve were released as well [5, 33].

By the third day of postoperative care, full range of motion at or beyond preoperative levels was observed. It was found that 98% of patients experienced improvement of scapular winging, and 92% of patients reported that they had satisfactory results [5].

Nerve Transfer Surgery

Functional restoration of the long thoracic nerve is possible using nerve transfer (i.e., neurotization) surgery [34-35, 5]. This procedure was performed on five candidates with upper-arm-type brachial plexus injuries resulting from motor vehicle accidents. Full brachial plexus exploration was performed with a sandbag under the affected extremity and patient supine in order to confirm C5 and C6 root avulsion. Subsequently, combined nerve transfers were executed in which the accessory nerve was transferred to the suprascapular nerve, a part of the ulnar nerve to the biceps brachii motor branch and the nerve to the long head of the triceps to the anterior branch of the axillary nerve [15].

The latissimus dorsi was retracted along the posterior aspect of the axilla to expose the thoracodorsal and long thoracic nerves. Once the long thoracic nerve was ascertained, intraoperative nerve stimulation was performed without contraction of the serratus anterior muscle. The thoracodorsal nerve was traced distally until its bifurcation into the medial and lateral branch. The segment that produced stronger muscle contraction of the latissimus dorsi upon intraoperative microelectrical nerve stimulation was chosen to be the donor nerve. In order to ensure that most of the serratus anterior muscle could be innervated, the long thoracic nerve was exposed and divided proximally along the chest wall. A nerve suture without tension or nerve graft should be performed since there is enough length. Patients used slings for three weeks after surgery. All subjects recovered full function of the serratus anterior and no obvious weakness of shoulder adduction was observed. However, three of the five patients had mild winged scapula after the surgery [15].

Nerve Grafting Treatments Based on Feasibility Studies

Based on cadaveric feasibility studies, it is possible to anastomose the accessory nerve with branches of the brachial plexus [16]. With this application in mind, the healthy contralateral long thoracic nerve may be able to restore function of the serratus anterior innervated by an injured long thoracic nerve. Since these are both motor neurons, the axons will regrow and the function of the damaged nerve can be recovered. For example, if the branch of the accessory nerve that innervates the lower trapezius is severed and reattached to the non-functional long thoracic nerve, reanimation of the serratus anterior is possible.

This use of the long thoracic nerve for reanimation of the targets of the ipsilateral facial nerve was deemed possible by a cadaveric study [19]. In the study, the long thoracic nerve contributions to the first and second digitations of the serratus anterior were preserved. The lower portion of the serratus anterior may have collateral innervations from the intercostal nerves [9, 36-37]. This implies that the distal segments of the long thoracic nerve can be dissected and transferred to alternate locations, while maintaining the function of the serratus anterior and thus preventing scapular winging. Since the long thoracic nerve possessed enough length to reach the extracranial facial nerve, it is evident that there is sufficient length to reanimate the trapezius if the long thoracic nerve is grafted to the accessory nerve.

Similarly, the long thoracic nerve can be utilized for contralateral neurotization to other nerves of the brachial plexus [18]. Indeed, this was noted to be physically possible if the long thoracic nerve is dissected distally and tunneled subcutaneously above the clavicle to the contralateral brachial plexus. It can be grafted to the suprascapular nerve near the upper trunk of the brachial plexus or to the musculocutaneous nerve [18]. These studies indicate that motor-to-motor nerve grafts can allow for reanimation of muscles originally innervated by the damaged nerve. Conceivably, this technique can be used as a therapy for scapula winging. The contralateral long thoracic nerve can be used to motorize an injured long thoracic nerve without loss of function of the original serratus anterior. Furthermore, only the apical region of the serratus anterior would have be to reanimated since the lower segment of this muscle may be innervated by intercostal nerves [9, 36-37].

The contralateral accessory nerve can be dissected and passed subcutaneously to the suprascapular and axillary nerves [16]. A posterior approach to the accessory nerve allows it to be extracted from its position deep to the trapezius muscle. 

The adequate length and diameter of the accessory nerve proved advantageous since an intermediate nerve graft need not be used to connect it to its new target nerves. In other words, direct anastomosis between the accessory nerve and the aforementioned nerves is possible [16]. Such features of the accessory nerve suggest a flexible and feasible application to the treatment of winged scapula when the condition is due to contralateral serratus anterior palsy. Although functional loss of the trapezius may also contribute to winged scapula, the functional serratus anterior and levator scapulae on that side may be able to maintain the position of the scapula [16].

The aforementioned techniques seem to be theoretically feasible for the treatment of serratus anterior palsy. It does not appear that there are any studies in the literature enumerating these procedures and their outcomes. Further quantitative research regarding rates of operative success and postoperative complications for these surgical procedures are thereby warranted.

Discussion

A variety of surgical treatments have been used over the years to rectify scapular winging. These can be put into the broad categories of wire fixation, muscle transfer, and nerve grafts. Wire fixation procedures include scapulopexy without arthrodesis and scapulothoracic arthrodesis. Muscle transfer procedures involve replacing the function of either the serratus anterior or trapezius and include procedures such as the pectoralis major transfer, split pectoralis major transfer, Eden-Lange procedure and the modified Eden-Lange procedure. Nerve graft repairs involve reinnervating muscle or repairing damaged nerves and include procedures such as nerve transfer surgery, nerve grafts, and microneurolysis of the long thoracic nerve.

The wire fixation techniques were generally successful, although there was a significant occurrence of surgical complications. Scapulothoracic arthrodesis is a static procedure used in the past to treat winged scapula secondary to trapezius muscle palsy [4]. Although it is less commonly used today, it seemed to have considerably high success rates with most patients experiencing less shoulder pain and greater range of motion [5, 13]. However, this technique can be fraught with undesired sequelae such as pneumothorax and pseudarthrosis. Surgeons proactively attempted to identify cases in which pneumothorax occurred to rectify the situation. Despite these efforts, the procedure remains risky and patients are advised to consider preoperative counseling before deciding to have this surgery [13].

Scapulopexy without arthrodesis is a similar procedure excepting that the semitubular fragment plate is not used to functionally fuse the scapula to the rib cage as described by Giannini, et al. [14]. It appears that this technique was more successful as all cases produced stable and persistent fixation of the scapula to the rib cage [14].

The muscle transfer techniques proved to be more diverse and variable with respect to the muscles used and the attachments made in the transfer. The pectoralis major transfer surgery produced fair results. Unfortunately, there were instances in which scar tissue and fraying of the fascia lata extension negated the effects of the muscle transfer. This resulted in recurrence of winging and pain [11]. A variation of this procedure known as the split pectoralis major transfer was more effective. Indeed, most subjects regained full range of motion of the shoulder and reported that the scar from the procedure was not cosmetically unappealing [4].

The Eden-Lange method is primarily used to correct winged scapula due to paralysis of the trapezius muscle. This technique was generally successful as patients reported pain relief and restoration of shoulder function [12]. However, once the modified Eden-Lange procedure was introduced, its popularity increased to such an extent that it replaced the original maneuver. Overall, it seemed that the modified procedure was advantageous since there was no residual winging in any patients [4].

Nerve grafting techniques are a more modern method of treatment for scapula winging and can involve combined nerve transfers. In the study described above, this method yielded variable results since some patients recovered full function of the serratus anterior and experienced no weakness, while others experienced mild winging even after the procedure [15].

Microneurolysis is a technique used to decompress the long thoracic nerve and thus restore function of the serratus anterior muscle. Overall, most patients experienced full range of motion of the shoulder and improvement of scapular winging after this procedure [5, 33, 38-39].

Finally, several prospective techniques for nerve grafting that may potentially be useful for treatment of winged scapula are emerging based on cadaveric feasibility studies. One such method involves anastomosis of the accessory nerve with branches of the brachial plexus and the use of the contralateral long thoracic nerve to reinnervate a paralyzed serratus anterior muscle.

## Conclusions

Full recovery from winged scapula, a condition uncommonly seen in clinics, is possible with early diagnosis and treatment. Surgical treatments have evolved over time, from wire fixation and muscle transfers to nerve grafts, each with advantages and common complications. Based on this review, the continued use of feasibility studies to investigate relatively new techniques such as nerve grafts may be one of the most significant ways in which medical literature can further contribute to the effective treatment of winged scapula. These studies could be used in surgical trials in order to identify ideal donors and target nerves for such procedures.
